# Mother’s freedom of choice and the rights of an unborn child: a comparison between the views of freshmen and senior medical school students

**DOI:** 10.6061/clinics/2016(10)03

**Published:** 2016-10

**Authors:** Marcelo Shigueo Yosikawa Motoki, Fabio Roberto Cabar, Rossana Pulcineli Vieira Francisco

**Affiliations:** Faculdade de Medicina da Universidade de São Paulo, Departamento de Ginecologia e Obstetricia, São Paulo/SP, Brazil

**Keywords:** Fetus, Ethics, Schools, Medical, Jurisprudence

## Abstract

**OBJECTIVES:**

To compare the views of freshman students with senior students of the Faculty of Medicine- University of São Paulo concerning the respect for the mother’s freedom of choice, the need to protect the unborn child, the proportionality between the mother’s freedom of choice and the protection of the unborn child, and issues related to legal abortion. To determine whether the medical knowledge acquired throughout the academic years can influence the views of medical students on these issues**.**

**METHODS:**

First- and sixth-year students of the Faculty of Medicine – University of São Paulo answered a questionnaire; the inclusion criteria were as follows: a first- or sixth-year student of the medical school and a signature on the free informed consent form. To compare the proportions, a chi-square or Fisher’s exact test was used. The significance level was set to 5%.

**RESULTS:**

Regarding the mother’s freedom of choice, in the case when a pregnant woman undergoes a cesarean section by means of a court order despite her intention to not have a cesarean, 55.7% of the first-year students have answered that the mother’s choice should be respected. Among the sixth-year students, only 28.9% believe that the mother’s intention should be considered (*p*<0.0001). With reference to the mother’s choice in connection with antiretroviral medication, 38.1% of the first-year students agreed that the mother’s intention should be respected, whereas 33% of sixth-year students believed that the mother’s intention should be respected (*p*=0.453).

**CONCLUSION:**

There was a tendency to consider the unborn child’s rights over the mother’s choice as students spent more time in medical school.

## INTRODUCTION

The legal protection of health assistance to any citizen is considered a fundamental right under the Brazilian legal framework. When health assistance is protected, the Law also covers life. To ensure the right to life (as set forth in Section 5, *caput,* of the Brazilian Federal Constitution, enacted on October 5^th^, 1988) with dignity (as set forth in Section 1, item III, of the Brazilian Federal Constitution, enacted on October 5^th^, 1988), under the terms and provisions of the Brazilian Federal Constitution, health assistance should be supported by the legal system in several ways. Thus, the right to health is considered a social right and has its exclusive section in the Constitution wording (Sections 196 to 200 of the Brazilian Federal Constitution enacted on October 5^th^, 1988).

An unborn child, or in Portuguese, “nascituro”, is a term derived from Latin that means "the one who shall be born" or one who was conceived but not born yet. The Brazilian and other Western legal systems protect the health rights of the unborn child exclusively through the criminalization of abortion (Sections 124 to 128 of the Brazilian Criminal Code). For other crimes for which health is protected, in this case, crimes that can be committed by anyone excluding the crimes that require a particular condition of the criminal (“crimes próprios” and “crimes de mão própria”, as defined in the Brazilian legal system; for example, infanticide), and those crimes in which the unborn child clearly could not be the victim (such as murder, inducement, instigation or accessory to suicide), the legal literature notes that the victim must be a living human person. In the crime of bodily injury, in the risk of venereal infection, or in the risk of serious disease infection, the legal literature is clear to state that the victim is the human person with life 1. The literature clarifies that an injury is any harm to the physical integrity or the physical or mental health of a person, including harm due to infectious diseases. In this respect, there is no provision similar to the provisions of Sections 130 and 131 of the Brazilian Criminal Code.

In Brazil, the Medical Code of Ethics 2, when referring to the relationship between the health professional and his/her patient, establishes the freedom of choice doctrine, providing that the physician is forbidden from starting any medical procedure without prior informed consent from the patient or his/her legal representative, except in the case of an imminent risk to life (emergency).

In Obstetrics, unlike all other medical specialties, the two lives (mother and unborn child) that are under medical care must be considered. Is the unborn a holder of his rights? Can a pregnant woman, to exercise her autonomy, put the unborn child’s rights at risk? Who has the duty to protect the unborn when the acts of a pregnant woman put the life and the future of the unborn child at risk?

Those questions do not have any answers yet and may be cause for discussions among all those who potentially can address this problem because we note a failure in the Brazilian legislative branch to provide legal and criminal protection for the unborn child.

In this article, we intend to compare the views of first-year and sixth-year students of a prestigious medical school to understand whether the medical knowledge acquired throughout the course of medical school can influence student’s views on the rights of the unborn.

## CASES AND METHODS

This prospective study compared the views of first-year students with sixth-year students of the Faculty of Medicine – University of São Paulo (FM), regarding the following:

- Respect to a mother’s freedom of choice;
- Need for protection of the unborn child;
- Issues related to legal abortion; and
- Proportionality between the mother’s freedom of choice and the protection of the unborn child.

First-year and sixth-year students of the FM were asked to answer a questionnaire with a copy attached to the Annex, and the cases were selected by applying the proposed criteria. The inclusion criteria were as follows: (i) a freshman or a sixth-year student of the FM and (ii) a signature of the free informed consent form. Responses to the questionnaire were excluded in situations where the responses were inadequate (i.e., questionnaire is blank – considered when more than 50% of the questions were not answered or when more than one alternative was marked on all questions) or when, after answering the questionnaire, a student underwent a motivated or unmotivated withdrawal from the study.

Efforts have been made to distribute a single questionnaire to each student in their first or sixth year at the FM. It was estimated that there are approximately 175 students in each class; estimating a loss of 40 to 50% of the population, it was expected that there would be 88 and 105 cases for each class.

The questionnaires and consent terms were delivered and collected personally by the researchers. Students were asked to complete the questionnaires during class with the permission of the professors.

Quantitative data are expressed as the means and standard deviations. To compare the proportions, the chi-square test or Fisher’s exact test was used. The significance level was set to 5%.

## RESULTS

In the current study, 201 cases were included, and 101 and 100 questionnaires were obtained from the first-year and sixth-year students, respectively. No cases were excluded.

To test whether the observed frequency was statistically equal to the expected frequency, considering the group data for age, sex and ethnicity for each year, the chi-square test was used. This comparison was possible because Fundação Universitária para o Vestibular (FUVEST) provides, on its website, the aforementioned information for freshmen each year. The results presented here show this is a homogeneous sample, which allows the assumption that this sample represents the entire group.

Among the first-year students, the average age was 19.52 years (standard deviation 1.90); 56.4% were men, 76% reported themselves as Caucasian, 11% Asian, 11% mixed ancestry and 2% black; among sixth-year students, the average age was 26.05 years (standard deviation 3.32); 58% were men, 73% reported themselves as Caucasian, 16% Asian, 8% mixed ancestry and 3% black. The other population characteristics are found in the records “sex”, “religion”, “marital status” and “children”.

I. Respect for the mother’s freedom of choice

Regarding respect for a mother’s freedom of choice, in the case of a pregnant woman who is submitted to a cesarean section by a judgment order, despite her intention to not have a cesarean, there was a statistically significant difference between the groups as follows: 55.7% of the first-year students answered that the mother’s intention should be respected. In contrast, only 28.9% of the sixth-year students believed that the mother’s intention should be respected (*p*<0.0001). Regarding a mother’s choice related to antiretroviral medication, there was no significant difference between the students’ answers as follows: 38.1% of the first-year students agreed that the mother’s intention should be respected, whereas 33% of sixth-year students believed that they should respect the mother’s intentions (*p*=0.453).

II. Need for protection of the unborn child

Regarding the protection of the unborn child, 75.5% of the first-year students said the unborn should have the protection granted by the law; among sixth-year students, 73.5% also said the same with no statistically significant difference between the groups (*p*=0.743).

It was questioned whether the unborn child was protected by civil law, and 84.4% of the first-year students said yes; among sixth-year students, 71.1% also said the same. Among those who had answered that the civil law does not protect the unborn child, 65.1% were sixth-year students, whereas only 34.9% were from the first year (*p*=0.002). A statistically significant difference was observed between the groups.

The same question was asked, but it was related to criminal law, and 64.9% of first-year students and 70.1% of sixth-year students answered that the unborn child is protected under criminal law. In the case of an HIV positive pregnant woman who did not take antiretroviral medication, students were questioned whether there should be a punishment. Of the first-year students, 31.1% said there should be a punishment for the mother, whereas 12% of the sixth-year students agreed. Among those who answered yes to this question, 70% were from the first year, whereas 58.7% were from the sixth year (*p*=0.001). Once again, a statistically significant difference was observed between the groups.

III. Issues related to legal abortion

When asked about the legalization of abortion, no differences could be found between the student groups as follows: 37.3% of the first-year students answered that it should be legalized in all cases; 4% of them answered that it should be prohibited in all cases; and 58.4% of them answered that it should be allowed in some cases. Among the sixth-year students, 44.4% of them said that it should be legalized in all cases; 4% of them said that it should be prohibited in all cases; and 53.5% of them said that it should be allowed in some cases (*p*=0.491).

When asked if students approved legal abortion indications, no difference could be found between groups as follows: 47.7% of the first-year students agreed with all indications; 4.7% of them did not agree with any indications; and 47.7% of them agreed with some indications; whereas 63.9% of the sixth-year students agreed with all indications; 4.1% of them did not agree with any indication; and 32% of them agreed with some indications (*p*=0.08).

IV. Proportionality between the mother’s freedom of choice and the protection of the unborn child

Considering the first-year students who had answered that criminal law does not protect the unborn, 75.9% of this group said there should not be any punishment for HIV positive pregnant women who did not take any type of medication; of those who had answered that criminal law provides protection, only 33.9% said that there should be punishment for pregnant women who did not take medication.

Regarding the sixth-year students who answered that criminal law does not protect the unborn, 93.1% of them replied that there should be no punishment. On the other hand, of those who had said criminal law provides protection, only 14.7% said there should be punishment.

## DISCUSSION

The protection of the right to health of individuals by the legislature can be observed in several branches of law, such as criminal protection considering people who commit acts against life or health of individuals or the mandatory obligation to supply medication or medical intervention when required to any person in need. Thus, the right to health assistance is constitutionally protected, which is closely connected to the right to life and the right to a dignified life.

The crime of putting someone’s life or health in danger may be committed by anyone, sick or not, with the effective exposure of the life or health to any danger. It is a crime that requires proof of the risk offered to the victim’s legal interest, and the victims must be, at least, determinable. The subjective element is composed of the offender’s serious criminal conduct and whether it is intentional 3.

The bodily injury offense is provided in its own section and consists of an effective injury act to the body or health of someone. Thus, a bodily injury is both the damage committed by somebody and the worsening of damage that arose from an existing situation 4.

The legal interest that is protected under the law is the safety of individuals, including their physical and mental integrity. The criminal is the same as set forth in Section 129, *caput,* and paragraphs 1, 2, 3 and 6. The law does not punish self-injury, unless it is committed for fraudulent purposes. The victim is the living human being. Hence, legal entities, animals and even the unborn or dead cannot be considered victims for the purpose of this crime 4.

Prado states that the crime aims to protect life; here, this is understood as the period between birth and death, ruling out the possibility of applying Section 129 of the Brazilian Criminal Code for the protection of intrauterine life as follows: “a victim is any living human being from the time birth has started” 3.

Teles has an opposite view as follows: “It is evident, then, the unborn under formation has its due bodily integrity that sustains life. If the latter is protected, then the former should be as well. And so, it is because society concerns the protection of human beings under formation not only against actions that may destroy them but also those that injure their bodily integrity or damage their health 5.”

Greco highlights the following relevant point related to the subjective element of bodily injury acts against intrauterine life: “If the criminal intended, as suggested by Teles, to offend the body or health of the fetus, he shall be claimed by the crime of bodily injury, and the only matter needed to be proved, at the time of criminal’s action, is the fetus was alive, a requirement for the offense configuration under the terms of law. Thus, the protection granted by the Brazilian Criminal Code starts from the moment where a new life born arises in the mother’s womb (…)” 1.

In the common context of the Western world, the protection of an unborn child’s health, a fetus under development with extra-uterine life potential, is provided exclusively by the criminalization of abortion, which can be a complete or partial criminalization as per the different legal systems; as an exception, the Spanish legal framework protects the health of the unborn by provisions other than abortion. Thus, Brazil is not alone in adopting provisions for the protection of the unborn child.

The positioning of other Western legal systems is close to the Brazilian system. While differing as to the permissibility of abortion, some protect health similarly.

However, in 1995, Spain enacted a reform to its criminal legal system that represents a new order with respect to the protection of the unborn child, and this protection remains in effect today despite the occurrence of other modifications to the criminal law. After the 1995 legislative reform of the Spanish Criminal Code, Sections 157 and 158 were added, related to, as said the current Title IV, “The injuries to the unborn child” (in free translation). The wording of Sections 157 and 158 of the Spanish Criminal Code is as follows:

“Section 157

Anyone that, by any means or process, cause to fetus an injury or illness that has serious consequences for its normal formation, or cause the same serious physical or mental harm, shall be punished with imprisonment of one to four years and relevant prohibition on exercising any medical profession or providing services of any type in a clinic or gynecological clinic, public or private, for between two to eight years.

Section 158

Anyone that, by gross negligence, commits the acts described in the preceding section, shall be punished with imprisonment of three to five months or a fine of six to 10 months.

When the events described in the previous article are committed by professional negligence, the penalty of specific disqualification from the exercise of the profession, trade or office for a period of six months to two years shall also be imposed.

The pregnant woman will not be punished under this provision.”

Section 157 provides that anyone who causes, by any means or procedure, injury or illness that harms the physical or mental formation of the unborn will be punished. Section 158 establishes a culpable provision (negligence) for the acts described in Section 157 and also establishes a provision for the professional who commits the crime in the exercise of his profession. Finally, pregnant women will not be punished in the event of Section 158.

From the analysis of those sections, a series of questions can be made. Firstly, what is meant by injury? These articles should be studied in connection with the provisions of Title III of the Spanish Criminal Code, which settles in Sections 147 to 156 the injuries to people. Section 147 shows an initial idea of injury:

“Section 147

1. Anyone that, by any means or process, cause another an injury that harms its bodily integrity or physical or mental health shall be punished as guilty of the crime of injury with imprisonment of six months to three years, provided that the injury to its health demands, along with an optional first aid, medical or surgical intervention. Simple surveillance or medical monitoring in the course of the injury is not considered medical treatment.

The same penalty will be imposed on anyone, that within a year, have conducted four times the action described in Section 617 of this Code.”

The Spanish legislature considered it more appropriate to include along the expression “lesion” any misconduct to physical integrity or physical and mental health of the unborn. A fetus, or an unborn child, for the purposes of Spanish criminal law, is the embryo that will grow from 57 days from the moment of fertilization until the moment of birth.

Autonomy, or freedom of choice, means personal self-governance and self-determination related to making decisions regarding someone’s life, health, physical and mental integrity and social relations. It calls for the existence of options, where the individual is free to choose, and requires that the individual will be able to act according to the decisions made. The respect for self-determination is based on the doctrine of human dignity, accepting the Kantian categorical imperative that states that a human being is a goal in itself.

Human dignity is one of the fundamental principles of the Federative Republic of Brazil, set forth in item III of Section 1 of the Brazilian Federal Constitution. Related to the human condition itself, dignity is a value from which all fundamental rights are derived. It is a moral value inherent to each person that reveals itself uniquely in the conscious self-determination and the responsibility of life and that brings an intention to respect by others. Dignity should be ensured by the legal system, so that only exceptional limitations on the exercise of fundamental rights can be enacted, but always considering the due respect people deserve as human beings.

This concept highlights how autonomy and freedom equate to dignity.

Brazilian law protects both the freedom of choice and self-determination doctrines. The Brazilian Civil Code (2002) provides that “no one shall be required to be submitted, at risk of life, to a medical treatment or surgical intervention.” So the patient, regardless of his or her medical condition, has his or her legal capacity granted by law, as well as his or her equal treatment related to rights and duties, and cannot be discriminated against on the grounds of age, race, sex, color, health status, nationality or religion. Villaça argues that when the clinical situation does not remove the ability to decide, physicians should respect the autonomy of the patient, even if it is an emergency 6.

Lately, the press made public the judgment issued by Judge Liniane da Silva, who after having taken the arguments of Prosecutors from Torres (State of RS), determined that a pregnant woman at the 42^nd^ week of pregnancy must be subjected to a cesarean section, in spite of her intention; this fact stimulated intense discussions involving several fields. In the context of medicine, medical interventions limit the pregnant woman’s liability, and its consequences were questioned.

Regarding the judgment, the dangerous precedent of judicial intervention was discussed, once the mother’s freedom of choice and the parents’ rights were not considered. The case involves the following two groups of individuals considered to be vulnerable: women and unborn children, requiring particular protection both by the health system and the justice system, vis-à-vis characteristics and discrimination.

In another similar situation related to the relevant confrontation of maternal autonomy and the rights of the unborn child, Cabar et al. mention the case of a child that had been born infected with HIV because her mother, who knowing that she was infected by this virus, had refused to take the drugs that could greatly reduce the risk of fetal infection during the prenatal period and birth 7.

How should this child be protected from a serious lethal disease? How then should the doctor proceed? Shall he respect the mother’s freedom of choice and put the unborn child’s life at risk?

For those reasons, if it is easy to conclude that the autonomy of the individual must be respected, it is unclear to infer whether the exercise of this autonomy could interfere, directly and negatively, on the health of others.

There are no studies in the literature that address those ethical and legal issues related to maternal autonomy and the rights of an unborn child. In this study, after the questionnaire and appropriate statistical analysis, it was possible to see significant differences between the views of the first and sixth-year medical school students.

Regarding knowledge of legal abortion permissions, most students, both in the first year (80.2%) and the sixth year (94%), have said they know them; of those who answered negatively, the majority (76.9%) were from the first year. This trend may be explained because as students progress in college, the matters related to abortion are presented, and, consequently, the students acquire knowledge on the subject.

Another possible conclusion concerning knowledge acquisition throughout the course was the result regarding the HIV positive pregnant woman who did not take antiretroviral to prevent the fetus from being infected. The success rate was higher in the group of sixth-year students as follows: 88% of them answered “no”. Among all the correct answers (“no”), 58.7% of them were from the sixth-year students (*p*=0.001).

Nevertheless, when asked whether criminal law protects the unborn, most students incorrectly answered “yes” and also equivocate themselves when asked if there would be punishment for pregnant women. In other words, the percentage of students who correctly answered both questions reached only 24%.

When asked about the approval of the judgment order to execute the cesarean section, in spite of the mother’s intention because there were risks for the mother and the unborn, there was a very large difference of views (*p*<0.0001) when analyzing the two groups. In the first-year students, most of them (55.7%) disagreed with the cesarean procedure because the mother’s intention should be respected. In the sixth-year students group, the opposite trend occurred because 71.1% of them were in favor of the judgment order, prioritizing the safety of the fetus, in spite of the mother’s freedom of choice. This supports the hypothesis that medical graduation influences the belief that unborn care is more important than women’s autonomy over their own bodies.

In the case of the HIV pregnant woman, there was a general agreement for not protecting the mother’s freedom of choice (61.9% of the first-year and 67% of the sixth-year students); that is, unlike the case of the cesarean section, first-year students chose to protect the fetus. Maybe this difference in view was due to differences in the maternal lack of comfort and the fetal consequences between the two cases. A cesarean section, such as any other surgery, involves inherent risks, such as infection, and when compared to natural childbirth, is a more invasive procedure. However, in the HIV case, the discomfort caused by taking the antiretroviral drugs and the disregard of the mother’s freedom would be less severe when compared to the consequences of not taking the drugs on the unborn child, who, from birth, would be infected with HIV, a pathology still very stigmatized by society and with no cure.

When the answers to the questionnaire on the judicial compulsory cesarean section were analyzed, in the HIV case, there was a consistency between the answers of the sample group as follows: most students said the integrity of the unborn was more important. When investigating the answers in the two separate groups, this consistency remained only among the first-year students as follows: considering the students who have answered that maternal autonomy should be respected when executing the cesarean section, the majority of the students was in favor of the mother’s decision to not take the antiretroviral drugs. Considering the students of the sixth year, regardless of an agreement or disagreement with the compulsory judicial caesarean section procedure, they had a tendency toward protecting the unborn in the HIV case. Throughout medical school, medical students gain more knowledge about this disease and the problems that it can cause both physically and psychologically. Therefore, it is natural that students with more advanced knowledge about HIV favor protecting the unborn child, even if they are not in favor of unborn rights in the C-section case. The same fact is observed when analyzing the answers to the question about the need for legal protection of unborn children and the need to protect the unborn when the mother does not want to take anti-HIV drugs. Among the sixth-year students, most of them chose to protect the unborn child, whether they were in favor of specific legislation to protect the lives of unborn children.

Unexpectedly, the same was not found in protecting the unborn child in the compulsory judicial cesarean section because most of the sixth-year students who replied that there should be legal protection to the fetus also said that the maternal autonomy should be respected.

Another interesting aspect was related to the student having a religion. It is known that many religions consider the unborn as a living being as follows: in spiritualism, the fetus already has a built-spirit that has previously been prepared to reach the world; in Catholicism, the theory of implantation also prevails, which states that life begins at conception. Among the first-year students, most of whom had no religion, 53.7% were in favor of the mother’s freedom of choice; among those who had a religion, 73.2% were in favor of protecting the unborn child. Among the sixth-year students, regardless of whether they had a religion, most of them were in favor of the unborn integrity (67%). Does this represent another instance of academic knowledge interference?

We underline that approximately 2/3 of students adequately fulfilled the questionnaire. This fact could have possibly biased the results because the other 1/3 of the students have not answered the survey.

It was not possible to assess the impact of years in medical school on the students’ choices. However, we imagine that it could have influenced the answers because there were differences between the choices made by first- and sixth-year students.

As emphasized above, there are no similar studies in the literature for a comparison of the results. This underlines the importance of this study and demonstrates the breadth of this theme. We conclude that the medical student, the one who will continuously address the theme of “respect for the patient’s freedom of choice” shows a tendency to support the rights of the unborn child over the mother’s choice as he/she advances throughout the medical school years. This conclusion is still not clear in our legal system.

## AUTHOR CONTRIBUTIONS

Motoki MS performed the study and analyzed the data. Cabar FR conceived, designed and performed the study. Francisco RP analyzed the data and revised the manuscript.
